# Random Forests for Global and Regional Crop Yield Predictions

**DOI:** 10.1371/journal.pone.0156571

**Published:** 2016-06-03

**Authors:** Jig Han Jeong, Jonathan P. Resop, Nathaniel D. Mueller, David H. Fleisher, Kyungdahm Yun, Ethan E. Butler, Dennis J. Timlin, Kyo-Moon Shim, James S. Gerber, Vangimalla R. Reddy, Soo-Hyung Kim

**Affiliations:** 1 School of Environmental and Forest Sciences, College of the Environment, University of Washington, Box 354115, Seattle, WA 98195, United States of America; 2 Department of Geographical Sciences, University of Maryland, College Park, MD, United States of America; 3 Crop Systems and Global Change Laboratory, USDA-ARS, Beltsville, MD 20705, United States of America; 4 Department of Earth and Planetary Sciences, Harvard University, Cambridge, MA 02138, United States of America; 5 Department of Organismic and Evolutionary Biology, Harvard University, Cambridge, MA 02138, United States of America; 6 Department of Forest Resources, University of Minnesota, St. Paul, MN 55108, United States of America; 7 Climate Change & Agroecology Division, National Institute of Agricultural Science, RDA, Suwon, Korea; 8 Institute on the Environment, University of Minnesota, St. Paul, MN 55108, United States of America; Instituto de Agricultura Sostenible (CSIC), SPAIN

## Abstract

Accurate predictions of crop yield are critical for developing effective agricultural and food policies at the regional and global scales. We evaluated a machine-learning method, Random Forests (RF), for its ability to predict crop yield responses to climate and biophysical variables at global and regional scales in wheat, maize, and potato in comparison with multiple linear regressions (MLR) serving as a benchmark. We used crop yield data from various sources and regions for model training and testing: 1) gridded global wheat grain yield, 2) maize grain yield from US counties over thirty years, and 3) potato tuber and maize silage yield from the northeastern seaboard region. RF was found highly capable of predicting crop yields and outperformed MLR benchmarks in all performance statistics that were compared. For example, the root mean square errors (RMSE) ranged between 6 and 14% of the average observed yield with RF models in all test cases whereas these values ranged from 14% to 49% for MLR models. Our results show that RF is an effective and versatile machine-learning method for crop yield predictions at regional and global scales for its high accuracy and precision, ease of use, and utility in data analysis. RF may result in a loss of accuracy when predicting the extreme ends or responses beyond the boundaries of the training data.

## Introduction

The ability to predict crop yield in response to climate variability at regional scale is crucial for developing agricultural policies, forecasting and analyzing global trade trends, and identifying effective adaptation strategies to climate change. Crop yield variability due to extreme weather events such as drought and high temperatures remains a major concern for farmers, governments, and markets, reinforcing the need for accurate and timely predictions of crop yield in an uncertain climate. In particular, year to year temperature fluctuations have been identified as the main source of uncertainty in crop yield predictions [1] while rapidly increasing global population is making food security an imminent challenge for many governments [2].

Two commonly used approaches for predicting crop yield responses to climate variability include process-based modeling and statistical modeling. Process-based crop models are powerful tools for crop yield predictions, particularly at the field scale, because they simulate physiological processes of crop growth and development in response to environmental conditions and management practices. However, due to intensive data and calibration requirements of process-based crop models, the use of detailed process-based crop models for timely predictions of crop yield at regional or global scale remains challenging [3]. On the other hand, statistical modeling estimates direct relationships between predictor variables (e.g., climate and soil factors) and crop yield in a given data set without considering the underlying processes in crop physiology and ecology. Statistical models can provide simple but reasonable predictions, provided that sufficient and reliable data were used for model training and that the predictions are made within the boundaries of training data. Statistical models are less dependent on field calibration data and may provide commonly used performance assessment measures useful for uncertainty analyses at regional scale [3]. Simple or multiple linear regressions (MLR) are commonly used statistical models for predicting crop yield [4, 5].

Statistical modeling methods based on machine-learning algorithms can provide alternatives to traditional regression approaches and overcome some of their limitations. These machine-learning techniques belong to the class of algorithmic approaches, which assume the data mechanism as a ‘black box’ or ‘gray box’. Machine-learning techniques have been used increasingly in recent years as niche-based classification modeling tools for predicting species habitat suitability in response to climate change [6, 7]. Random Forests (RF) is a non-parametric advanced classification and regression tree (CART) analysis method that has been adopted widely in many scientific fields, including for predicting suitable habitat of various plant and animal species [8–11] and gene expression interpretation [12–14]. A majority of applications of RF have been focused on its utility as a classification tool with only limited studies exploring its regression capabilities for predicting ecosystem or crop productivity [15–17]. Several studies have pointed to a number of promising advantages as well as drawbacks of RF as a regression tool over traditional regression models [18–20]. To date, RF regression applications in the fields of agronomy and crop science remain scarce, with few exceptions [15].

**Fig 1:**
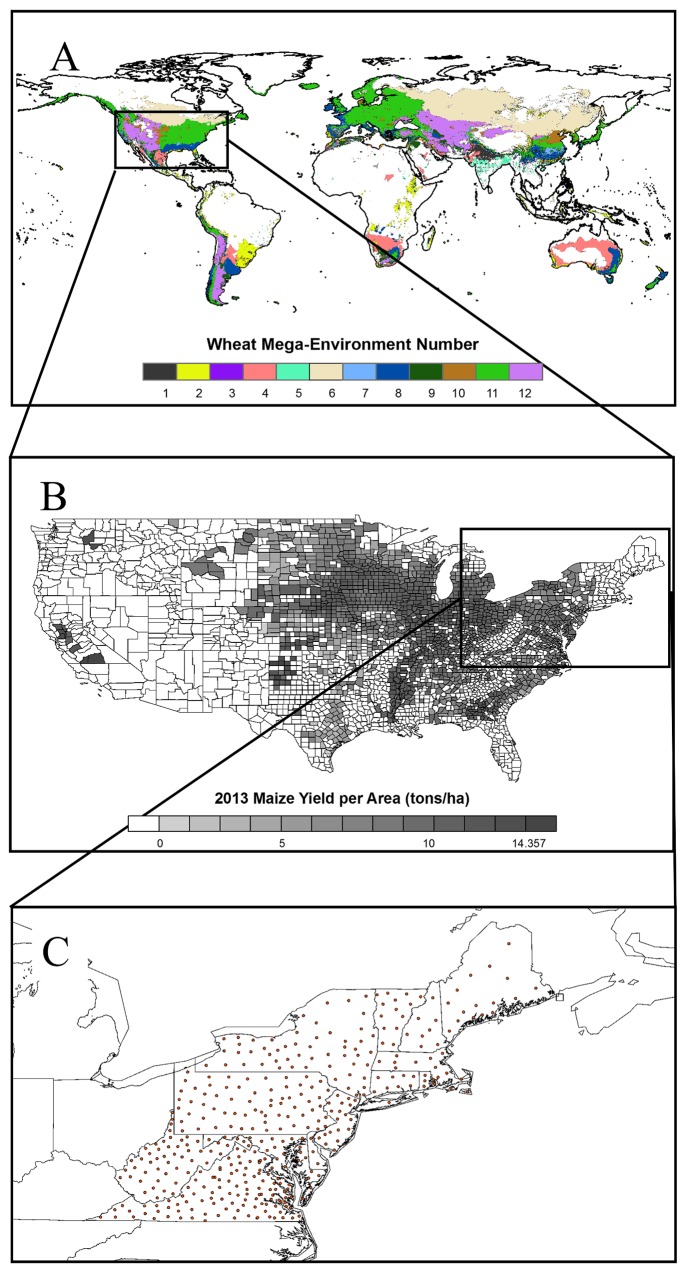
**Study regions: global wheat mega-environments (A), US maize producing counties (B), and northeastern seaboard region (NESR) (C).** All 12 wheat mega-environments are shown with different colors (A). Maize grain yield by the US counties in 2013 surveyed by USDA-NASS is visualized using different shades with darker shades representing higher yields (B). The NESR includes 433 counties of Connecticut, Delaware, Maine, Massachusetts, New Hampshire, New Jersey, New York, Pennsylvania, Rhode Island, Vermont, Virginia, and West Virginia. The red dots indicate the location of the data points, where weather stations exist. Point type data was used for this region (C).

**Fig 2:**
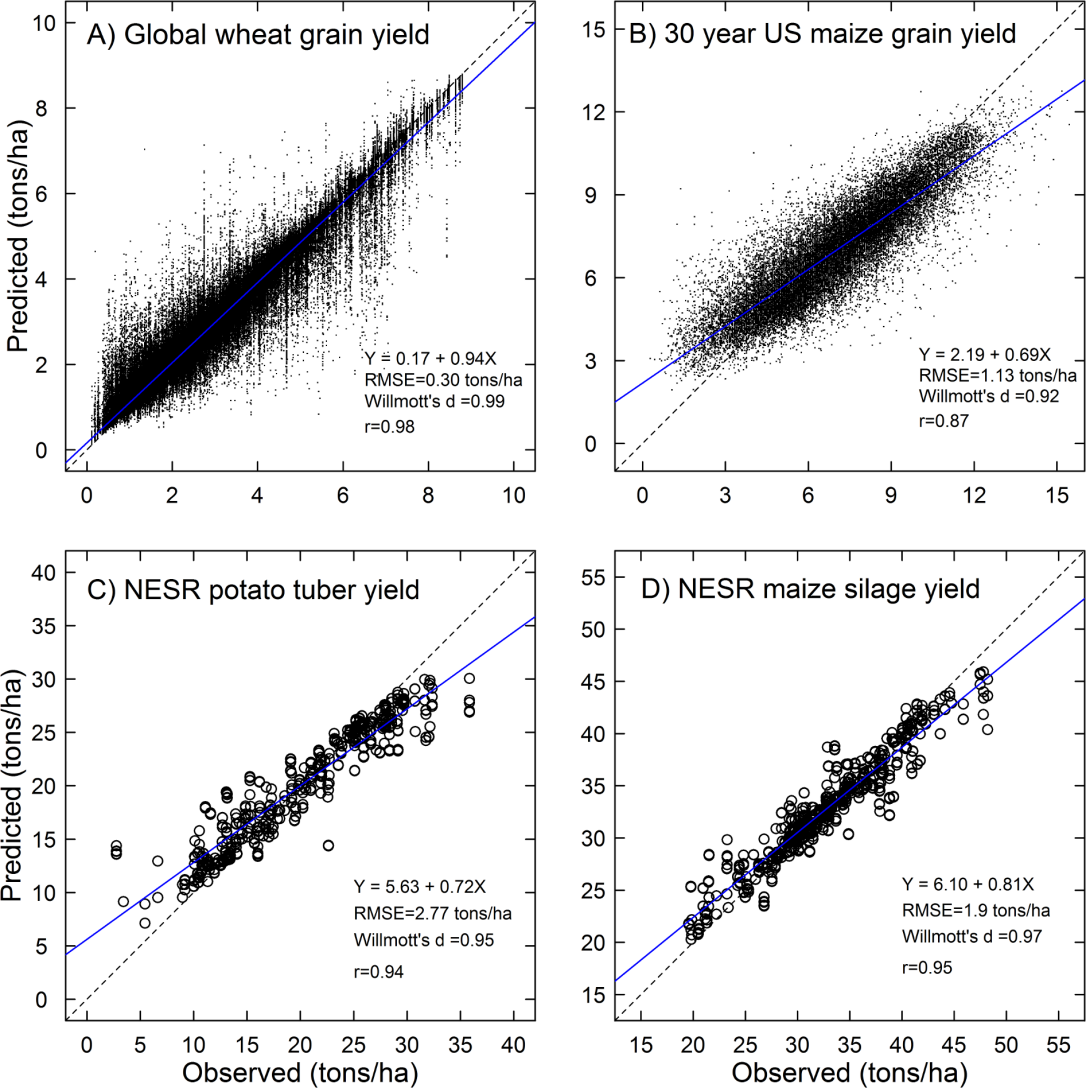
Random Forests model performance for test datasets. Observed vs. predicted plots are shown for four case studies: (A) global wheat grain yield, (B) US maize grain yield over 30 years, (C) potato wet tuber yield in northeastern seaboard region (NESR), and (D) maize silage yield in NESR The dashed lines indicate 1:1 relation and the solid line represents linear regression between the observations and predictions made for test datasets. The linear regression equation for the solid line is provided along with *RMSE*, *EF*, *d*, and Pearson’s *r*.

**Fig 3:**
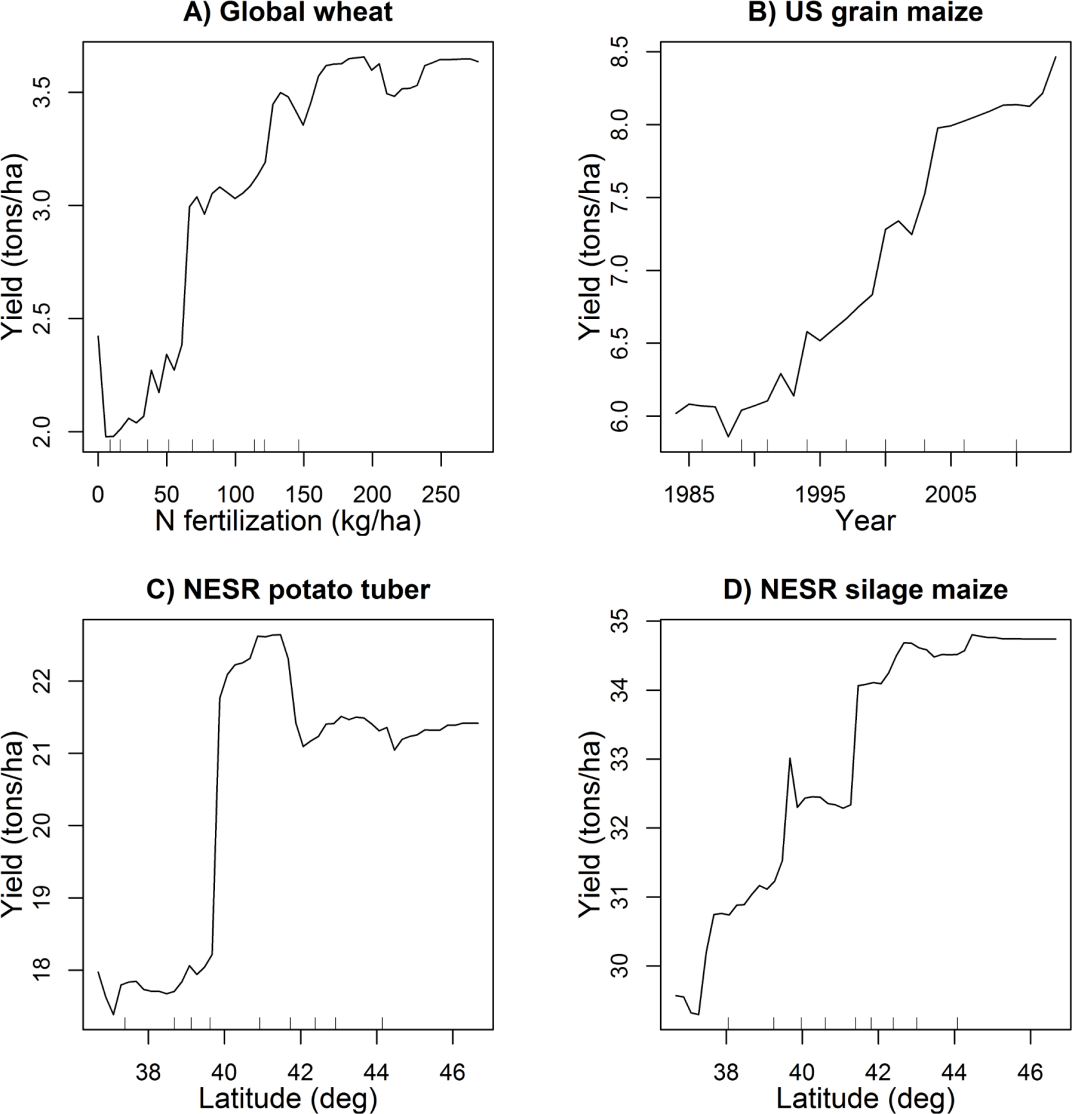
Partial dependence plots for the top ranked predictor variable from variable importance measures of Random Forests models. (A) N fertilization rate (NFERT) in global wheat grain yield predictions, (B) year (YR) in the 30-year US maize grain yields, (C) Latitude (*lat*) for potato wet tuber yields in northeastern seaboard region (NESR), and (D) *lat* for maize silage yield in NESR. The *Y*-axis of each plot indicates the average of all of the possible model predictions for the *X* predictor value. The *X*-axis hash marks indicate deciles.

The objective of this study was to evaluate the performance of RF regression using MLR as a benchmark for global and regional crop yield predictions for three staple crops: wheat (*Triticum aestivum*), maize (*Zea mays*), and potato (*Solanum tuberosum*). Specifically, we applied RF to predict: 1) grain yield of wheat at the global scale that included all regions designated as mega-environments (ME) defined by the International Maize and Wheat Improvement Center (CIMMYT) (Fig 1A), 2) county-level maize grain yield in the United States for a 30 year period (Fig 1A), and 3) yield of potato tuber and silage maize in the Northeastern Seaboard Region (NESR) consisting of thirteen states from Maine to Virginia of the United States (Fig 1B).

## Materials and Methods

The RF models were trained to predict crop yield using multiple biophysical variables as predictors (Table 1). Environmental variables included climate, soil, photoperiod, water, and fertilization data. The same data were used for training MLR models for benchmarking purposes. The RF algorithm intrinsically set aside partial data for its own internal validation, called out-of-bag (OOB) data. However, to ensure a fair and conservative comparisons between RF and MLR, we used only a random half of each dataset (i.e., wheat, maize grain, potato, silage maize) for training (‘training dataset’) both RF and MLR models. The other half that was not used for training was then used as the ‘test dataset’ to validate and compare performances between the RF and MLR models. This process ensured that identical data points were available for training and independent testing using the data points not included in training.

**Table 1:** The predictors used for random forests (RF) and multiple linear regression models. Rank corresponds to a variable importance measure determined by RF models for each dataset.

Global wheat and US maize grain yields			Importance Rank		NESR potato tuber and silage maize yields			Importance Rank	
Variable	Abbreviation	Unit	wheat	maize	Variable	Abbreviation	Unit	potato	maize
Averaged monthly temperature	AVT	°C	8	9	Bulk density (soil)	*bulk*	g/cm^3^	9	9
Annual evapotranspiration	EVA	mm	2	6	Clay content (soil)	*clay*	-	7	2
Summer solstice day length	DAYL	hour	4	7	Hydraulic conductivity	*hyd*	cm/day	8	5
Maximum monthly temperature	MAX	°C	7	3	Average maximum daily temperature	*maxt*	°C	6	4
Mean coldest quarter Temperature	MCQ	°C	9	11	Average minimum daily temperature	*mint*	°C	5	7
Minimum monthly temperature	MIN	°C	10	8	Annual precipitation	*precip*	mm	4	8
Mean warmest quarter temperature	MWQ	°C	6	10	Averaged seasonal radiation	*rad*	MJ m^-2^ d^-1^	2	3
Nitrogen fertilizer application rate	NFERT	kg/ha	1	2	Saturated water content	*sat*	%	10	10
Growing season precipitation	PRE49	mm	3	4	Irrigation	*water*	1 = Yes; 0 = No	11	11
Annual precipitation	PRECI	mm	5	5	Latitude	*lat*	degree	1	1
Year (US maize only)	YR		-	1	Elevation	*elev*	m	3	6

### Global wheat yield data

Global wheat grain yield were determined for regions included in wheat mega-environments (MEs) defined by CIMMYT who defines each mega-environment as a broad region where crops share similar biotic and abiotic stresses, cropping system requirements, and consumer preference in the world [21]. See more details and global distribution of wheat MEs at: http://wheatatlas.org/megaenvironments. We employed the wheat mega-environment approach because it specifies global extent and limits of wheat production throughout the world.

The wheat yield data used for model training and testing were obtained from a published source [22] (http://www.earthstat.org/, accessed Jan 14, 2016). The dataset includes an average wheat grain yield (tons/ha) centered on year 2000 based on census data mostly collected from 1997 to 2003 on five arc-minute resolution. We used raster data with no spatial gaps inside the extent with an assumption that the entire area within a grid cell has the same value. The raster data layers were transformed into point data and spatially joined to each other based on the centroid of each grid cell by a Geographical Information System (GIS) program, ArcGIS 10 [23].

Four climate variables including monthly precipitation, maximum temperature, minimum temperature, and mean temperature for current (2000) normal, and seven additional biophysical variables used for training RF and MLR models for global wheat yield predictions (see Table 1). The climate data were obtained from WorldClim (http://www.worldclim.org/current, accessed Feb 19, 2016), The climate dataset is based on the average of observed climate records from 47,554; 24,542; and 14,835 weather stations for precipitation, maximum/minimum temperature, and mean temperature, respectively [24]. The averaged climate observations were interpolated by the software ANUSPLIN [25] with the National Aeronautics and Space Administration (NASA)’s Shuttle Radar Topography Mission (http://www2.jpl.nasa.gov/srtm/, accessed Feb 19, 2016) elevation dataset on a 30 arc-second resolution grid (often referred to as 1km square resolution) to make data layers for the entire world. Mean minimum coldest quarter temperature (MCQ), mean minimum warmest quarter temperature (MWQ), annual average temperature (AVT), annual precipitation (PRECI), accumulated precipitation during April to September (PRE49), annual minimum monthly temperature (MIN), and annual maximum monthly temperature (MAX) were then calculated from the source climate data using ArcGIS 10 (See Table 1). All of the climate data were joined with the wheat yield data after aggregating it to five arc-minute resolution using the means to match the resolution. The summer solstice day length (DAYL), nitrogen fertilizer application rate (NFERT), and estimated annual evapotranspiration (EVA) were used as additional variables. Summer solstice day length was incorporated into the model to account for the influence of photoperiod to crop yield, especially considering the high photoperiod sensitivity of the wheat cultivars. In addition, nitrogen fertilization (NFERT) was used to evaluate the impact of the agricultural management and the soil nutrient level. These data were from the crop-specific nitrogen fertilizer application rate map on five arc-minute resolution [26]. The map averaged 7 years (1997–2003) of fertilizer application rate in kg/ha based on the data from national statistical bureaus, FAO reports, and national-level fertilizer industry associations for a reference year: 2000. Estimated evapotranspration (EVA) was also included as a predictor. The evapotranspiration amount (mm/year) data is from the ‘Global map of yearly actual evapotranspiration’ from FAO GeoNetwork at five arc-minute resolution, and is based on a simple water-balance model (http://www.fao.org/geonetwork/srv/en/metadata.show?id=37233, accessed Feb 19, 2016).

### 30 year US maize grain yield data

We used county-level crop harvested area and yield data for maize from USDA-NASS (United States Department of Agriculture–National Agricultural Statistics Service) annual surveys for a thirty-year period of 1984–2013 [27] Crop areas and yields were converted to metric units using yield conversions from the USDA Economic Research Service. We used United States Historical Climatology Network daily weather data (temperature and precipitation) obtained from the Global Historical Climatology Network [28, 29]. These daily weather data were interpolated to county centers using a Delaunay Triangulation [28]. From the daily weather data, we calculated the same climate variables as used for wheat (i.e., MCQ, MWQ, AVT, PRECI, PRE49, MIN, MAX) for each year and county. The same additional variables (i.e., NFERT, EVA, and DAYL) used in the global wheat model were also included as input variables for predicting US maize grain yields. In addition, year (YR) was included as a predictor to account for temporal variability due to technological advances such as genetic and management improvements. Grain yield predictions were made for individual years for all counties, and used for comparing model performance between RF and MLR.

### Northeastern seaboard region (NESR) potato tuber and maize silage yield data

We used point data corresponding to spatial modeling units (MU), defined as field-scale polygons with homogeneous properties [30], for the NESR potato and silage maize models. Variables in the NESR dataset are denoted using lower-case italic acronyms (e.g., *maxt*) to distinguish from the variables used for ME6 wheat and US grain maize predictions. We used 1044 and 1512 MUs located in 435 counties in the NESR for modeling potato and silage maize yields (Fig 1B). Briefly, each county included approximately three modeling units. The United States Department of Agriculture (USDA) National Agricultural Statistics Service (NASS) census data for county level crop yield and harvested area [31] and NASS cropland data layer products [32] were used. Harvested area and crop yield for each point were obtained by averaging the last four years on record (1987, 1992, 1997, and 2002). The yield for each crop in the dataset is defined as harvested silage weight for maize and dry tuber weight for potato.

Annual precipitation (*precip*), average maximum/minimum daily temperature (*maxt*, *mint*), and average seasonal radiation (*rad*) were used as the weather predictors for RF and MLR model for potato and silage maize in NESR. The weather data were obtained by averaging observed weather data between 1960 and 2009 from the National Climatic Data Center which has 378 weather stations within the NESR. Saturated water content (*sat*), hydraulic conductivity (*hyd*), clay content (*clay*), bulk density (*bulk*), and irrigation (*water*) were used as non-climatic predictors for the models. The U.S high-resolution soil profile database (SSURGO) at a 1:24,000 scale was used to obtain the soil data. The soil data are averaged over all horizons in the soil profile. The binary factor variable ‘water’ indicated whether the cropping area around the MU was irrigated or not. See Resop et al. [30] for more detailed description of the NESR data.

### Random Forests (RF) predictions

We trained and applied RF, a binary tree based machine-learning method, to predict yields of three crops: wheat, maize, and potato. RF can be used for both classification and regression purposes, and the scope of our study is to use it as a regression tool. Briefly, to train RF models, many classification and regression trees (CARTs) are grown with a ‘random’ subset of predictors without pruning, and the ‘forest’ of CART is averaged. Source data for model training are bootstrapped to make various subsets to generate a large numbers of trees randomly. Predictor variables are evaluated by how much they decreased node impurity when they selected for the splits or how often they make successful predictions in the forest of CARTs. Node impurity is defined as mean square error (MSE) of the node in RF regression. See Breiman [18] for more details on the RF algorithm. We used the statistical program R [33] along with the package ‘randomForest’ with the following settings (mtry = 3, ntree = 300, nodesize = 5) [34]. Two variable analysis tools available from the package were used for analysis: variable importance and partial dependence plots. First, mean decrease accuracy (“%IncMSE”) used as a measure of variable importance. The %IncMSE plot shows the mean increase of MSE in nodes that use a predictor in the model, when values of the predictor are randomly permuted. The OOB data are used to compare average node MSEs before and after the permutation. The second analysis tool, partial dependence plot, shows how the RF model predictions are influenced by each predictor when all of the other predictors in the model are being controlled. The *Y*-axis value of a partial dependence plot is determined by the average of all of the possible model prediction with the dataset when the value of the objective predictor is *X*.

### Multiple linear regression (MLR) models

Multiple linear regression models (MLR) were constructed using stepwise variable selection method based on exact AIC (Akaike information criterion) using R ‘lm’ and ‘stepAIC’ functions for benchmarking the performance of RF predictions [33, 35]. The same predictors used in the RF models were included to build the MLR models. See Table 1 for variable definitions used in RF and MLR models. In addition, the quadratic terms of three variables (NFERT, PREC49, DAYL) were included when they improved the model performance significantly.

### Model performance evaluation and inter-comparison

Four methods were used to evaluate and compare the model performances: 1) root mean square error (*RMSE*, Eq [1]), 2) Nash-Sutcliffe model efficiency (*EF*, Eq [2]), 3) index of agreement known as Willmott’s *d* (*d*, Eq [3]) [36], and 4) observed vs. predicted plots. These are measures commonly used for agricultural systems and crop models [37, 38].

$$RMSE=\sqrt{\sum_{i=1}^{N}{\left(y_{i}-{\hat{y}}_{i}\right)}^{2}/N}$$ [1]

$$EF=1-\frac{\sum_{i=1}^{N}{\left(y_{i}-{\hat{y}}_{i}\right)}^{2}}{\sum_{i=1}^{N}{\left(y_{i}-\bar{y}\right)}^{2}}$$ [2]

$$d=1-\frac{\sum_{i=1}^{N}{\left(y_{i}-{\hat{y}}_{i}\right)}^{2}}{\sum_{i=1}^{N}{\left(\left|{\hat{y}}_{i}-\bar{y}\right|+\left|y_{i}-\bar{y}\right|\right)}^{2}}$$ [3]

Where, *y*_i_ represents the observations in the test data sets, *ŷ*_i_ the predictions, and $\bar{y}$ is the observation mean in each test dataset. Briefly, *RMSE* is a measure of deviations between the observations and model predictions. *EF* is a model skill score in comparison with the observation mean; a perfect model will produce *EF* = 1 while a model with the same predictability as the mean will have *EF* = 0. A value of *EF* < 0 is also possible with no lower boundary [37, 39]. Willmott’s *d* value varies from 0.0 (poor model) to 1.0 (perfect model) [36, 37]. In addition, an observed vs. predicted plot was made to visualize the model performance. A simple linear regression line was drawn over the plot to compare the accuracy of the model predictions. All performance measures (i.e., *RMSE*, *EF*, *d*, and observed vs. predicted comparisons) were assessed using the model predictions made for test datasets set aside for validation and evaluation purposes only. That is, no training data were included for assessing model performance.

## Results

### Global wheat yield predictions

RF successfully predicted global scale wheat yield when compared against the test data that had not been included in model training. The RF model explained 96% of yield variance with a good agreement between the predicted and observed values in the test data (Table 2; Fig 2A). The *RMSE* of the model was 0.32 tons/ha, which is 11.9% of the average observed yield, *EF* was 0.96, and *d* was 0.99. The performance of a MLR benchmark was less satisfactory: *RMSE* = 1.32 tons/ha which is 49.2% of the average observed yield, *EF* = 0.31, and *d* = 0.68) (Table 2). The comparison between the observed and predicted (Fig 2A) indicates that RF model predictions corresponded highly with the observations with a slope of 0.94 and Pearson’s *r* = 0.98. Overall, RF clearly outperformed MLR in all measures of model performance for global wheat yield predictions (Table 2).

**Table 2:** Random Forest (RF) and multiple linear regression (MLR) model performance evaluation statistics. See text for detailed description and equation of each statistic shown in the table.

Crop	Scale	RF			MLR		
		*RMSE* (tons/ha)	*EF*	*d*	*RMSE* (tons/ha)	*EF*	*d*
Wheat	Global	0.32	0.96	0.99	1.32	0.31	0.68
Maize (grain)	U.S.	1.13	0.76	0.92	1.93	0.30	0.67
Potato	NESR	2.77	0.75	0.95	5.62	-0.87	0.73
Maize (silage)	NESR	1.90	0.85	0.97	4.54	-0.41	0.75

Variable importance measures of the RF model revealed that NFERT was the most influential variable followed by EVA, PREC49, and DAYL (Table 1). Climate variables were ranked lower in their relative variable importance. The partial dependence plot for NFERT indicates that global wheat yield begins to saturate at N fertilization rates between 150 and 200 kg/ha when the effects of other factors were excluded (Fig 3A).

### 30 year US maize grain yield predictions

The RF predictions for 30 year US maize yield by county were satisfactory with an *RMSE* of 1.13 tons/ha, which is 16.7% of the average observed yield, *EF* was 0.76, and *d* was 0.92. The performance of a MLR benchmark was less satisfactory: *RMSE* = 1.94 tons/ha which is 28.7% of the average observed yield, *EF* = 0.30, and *d* = 0.67) (Table 2). The comparison between the observed and predicted (Fig 2B) indicates that RF model predictions correspond quite well with the observations with a slope of 0.69 and Pearson’s *r* = 0.88. Similar to the global wheat predictions case, RF clearly outperformed MLR in all measures of model performance for US maize yield predictions for 30 years (Table 2). Variable importance measures of the RF model for 30 year maize grain yield in the US revealed that year (YR) was the most important variable to influence yield variability followed by N fertilizer rate (NFERT), maximum temperature (MAX), and growing season precipitation (PREC49) (Table 1). Other climate variables were ranked lower in their relative variable importance. The partial dependence plot for YR illustrates a clear trend of yield increase (approx. 2.5 tons/ha) over the 30 year period in US maize (Fig 3B); this increase was accounted for by year only and not other factors included in the model.

### Potato tuber and maize silage yield predictions in NESR

Similar to the global wheat and US grain maize case, the RF showed substantially improved performance measures for potato tuber and maize silage yield predictions in NESR compared to the MLR (Table 2, Fig 2C and 2D). For potato tuber yield, the RF model produced an *RMSE* of 2.77 tons/ha (13.9% of the average observed yield), *EF* of 0.75, and *d* of 0.95 while MLR model resulted in an *RMSE* of 5.62 tons/ha (28.1% of the average observed yield), *EF* of -0.87, and *d* of 0.73.

For maize silage yield, the RF model showed an *RMSE* of 1.90 tons/ha (5.8% of the average observed yield), *EF* of 0.85, and *d* of 0.97 while the MLR model had an *RMSE* of 4.54 tons/ha (13.8% of the average observed yield), *EF* of -0.41, and *d* of 0.75.

Variable importance identified by the RF model differed somewhat between potato and maize (Table 1). The latitude (*lat*) variable was deemed most important determining both potato tuber and maize silage yield in NESR followed by *rad*, *elev*, *precip*, and *mint* for potato and by *clay*, *rad*, *maxt*, and *hyd* for silage maize (Table 1). Both potato tuber and silage maize showed higher yields at northern latitude locations (Fig 3C and 3D).

## Discussion

Our results illustrate that RF regression is highly effective for global and regional scale crop yield predictions. The RF models outperformed MLR models in all crops and scales we tested. Although RF has been used widely as a classification algorithm for species distribution and habitat suitability modeling in ecological studies in recent years [6, 11] to date few studies have explored its regression capabilities for crop yield or primary productivity studies in agriculture and ecology. Our results demonstrate that RF regression has many merits that are desirable for predicting complex crop responses in agricultural systems.

The RF algorithm intrinsically separates a random subset of data for performance testing from calibration data by using only the remaining set of data for model training. Therefore, splitting data for training and testing purposes is likely a redundant procedure when applying RF for crop yield modeling and its performance may increase as more data are included for training. For example, the RF model performance improved when we increased the size of training data used for potato tuber and maize silage predictions in NESR whereas MLR model performance did not improve (data not shown). Nevertheless, we separated training and test datasets in order to ensure that the same data points were used for training and testing between RF and MLR models. In practice, including more data for model training is likely to improve the predictability of RF regression.

Overall the RF model performed best (e.g., *EF* = 0.96) for predicting out of sample global wheat grain yield than any other cases we tested. However, the RF model performance was also highly satisfactory in other applications when applied for predicting US maize grain yields over 30 years or in NESR with much smaller datasets to train the model (Table 2; Fig 2). We note that the high performance for global wheat yield may be a result, in part, due to spatial autocorrelation in yields between grid cells belonging to identical political units; this feature of the gridded yield data limits evaluation for both RF and MLR.

In addition to its predictive capability, RF can also provide useful information about the variable importance and dependence. The variable importance rank and the partial impact of the variable on the response can be evaluated for the purpose of systems analysis [12, 40–42]. We used a commonly used variable importance measure: mean decrease accuracy (%IncMSE in r randomForest output) to identify the most influential variables determining crop yield in the regions we tested (Table 1). The partial dependence plots are useful for assessing the relationship between each predictor and the response variable [43, 44]. The variable importance measures and partial dependence plots revealed unique responses with respect to crops and regions. For example, variable importance output and the partial dependence plots show that NFERT was the top predictor of global wheat yield and US maize yield with a saturating response to increasing fertilizer (Fig 3). For 30-year US maize yield predictions, the model performance improved substantially when YR was included in the predictors; EF was 0.76 with YR and 0.64 without YR included. The temporal yield trends due to technological changes such as genetic improvements, agronomic intensification, pesticides and herbicides uses, and management practices have been well-documented [26, 45] and the RF model identified this trend as the most influential variable that was responsible for over 2 tons/ha increase over the 30 year period in determining maize grain yield in the entire US (Fig 3B). With the NESR cases, latitude (*lat*) was found to be the most influential variable to determine both potato tuber and maize silage yield (Fig 3C and 3D). Clay content was ranked as the second most important variable for maize silage yield in NESR as yield decayed with increasing clay content (data not shown) indicating drainage may be a key factor to achieving high silage corn yield in the NESR. The partial dependence plots for other variables showed relatively small effects with complex patterns that may not be readily explained by known physiological or agronomic responses indicating limitations of the analyses solely based on RF model outcomes.

Our results showcase the utilities of RF regression for crop yield predictions. Multiple advantages may exist for using RF regression over other approaches including traditional MLR when predicting crop yield responses. First, RF regression models have been shown to outperform traditional MLR models in explaining the variability in data [46] and our results strongly support that this is the case for crop yield predictions. Note that there could be many ways to improve MLR model performance such as including additional predictor variables. For example, there is evidence that the inclusion of extreme temperatures could further improve the performance of MLR models [47–49]. We did not include additional variables only to MLR models to maintain the predictors between RF and MLR comparable. The high performance of RF is likely more evident when the response is a result of complex interactions between multiple predictors as in crop systems where interactions among biophysical, ecological, physiological, and management factors can complicate modeling. Second, the RF regression has advantages when predictor or explanatory variables are highly correlated (e.g., temperature derived variables) [19, 20]. Many predictors of crop production, such as climate, management, and soil, are often highly correlated with and within each other and may have multi-collinearity. Variable collinearity can be a critical problem in traditional regression models that are derived from linear regression. RF uses the single best variable when it splits responses at each node of decision trees and averages the predictions of the trees in the forest to make a multi-dimensional step function. This means that even if multiple variables are correlated and drive the response similarly, only one of them can affect the RF regression model at a time. Third, RF algorithms can use multiple types of predictors in a model more easily than traditional multiple linear or non-linear regressions can [20]. Available data for crop yield prediction vary by type and collection methods. For example, temperature and precipitation data are quantitative and continuous while soil orders and crop cultivars are categorical. Sometimes, continuous data recorded is in a categorical form, such as high, medium, low content of soil organic matter. RF is an ensemble of decision trees, which consist of binary nodes that split the response. At every RF node, any type of splitting of predictor variables, such as continuous and categorical, is evaluated and selected for the split under the same standard: how well the given variable can split the response.

Limitations of the RF regression for crop yield modeling are also identified. As found with partial dependence measures of low importance variables (data not shown), the behavior of the RF model may be less intuitive to interpret than traditional regression models because its algorithm consists of an ensemble of a large number of decision trees that may not be fully described mechanistically. In addition, the RF algorithm may overfit data [18, 50]. In general, statistical models have the potential of overfitting issues within the range of training data. With RF, only the values included in training data are used for splitting regression trees and thus lumping the predictions for the conditions outside the range of training data. This means making predictions beyond the training data range is impractical because of inherent inability to extrapolate to data dimensions (e.g., future climate) where no training has been done. For predicting crop yield in the future scenarios, this limitation of RF regression could be critical, since at least some part of the current crop field is expected to have new and more extreme environmental conditions in the future that did not exist in the past and present data domains. In practice, the model training dataset should be selected to cover as wide a range as possible for most critical predictor variables. An approach to overcome the limitations associated with predicting novel extremes may be to train RF to learn the novel responses by coupling it with process- or MLR models that are capable of modeling crop responses in extreme conditions where observational data are sparse.

The balance of the variable variance distribution also needs to be considered in RF modeling. It is known that if the number of positive and negative response in the model training dataset is unbalanced, RF classification models overestimate the higher numbered responses [18]. The splitting and averaging algorithm of RF regression may result in underestimation of the marginal responses in the range where data points are scarce in extreme ends (see Fig 2). This response distribution balance can affect RF based crop yield predictions. Although a sufficient sample size can minimize this problem, an RF model’s predictive power for a future period can be compromised if the response domain is not conserved in the future scenarios. All of the RF models in this study showed an overall trend of underestimation above the mean and overestimation below the mean (Fig 2) because the marginal yield points were either underestimated or overestimated. Having a sufficiently large training dataset with balanced predictor variance distributions is likely to help minimize this issue; this is illustrated in the global wheat case where both accuracy and precision were improved compared to the other cases (Fig 2).

## Conclusions

This study evaluated the efficacy of RF regression using MLR as a benchmark to model complex yield responses of wheat, grain maize, potato, and silage maize at global and regional scales. The RF algorithm has many advantages to regress complex crop systems, but is not yet being widely used in this field. We demonstrated that RF provides superior performance in predicting yield of all crops and regions tested. The result of this study shows strong potential for the implementation of an RF algorithm as an alternative statistical modeling method for crop yield predictions. It should be noted that RF has the risk of overfitting data for the conditions where training data were concentrated while its accuracy can diminish where training data were sparse. Similarly, applying RF regression to extrapolate outside training data dimensions should be avoided. In summary, our results support that RF regression can be an effective tool for predicting crop yield at the global and regional scale with cautious selection of a training dataset that includes a wide range of predictor variability.
